# Parasite Mitogen-Activated Protein Kinases as Drug Discovery Targets to Treat Human Protozoan Pathogens

**DOI:** 10.1155/2011/971968

**Published:** 2011-02-27

**Authors:** Michael J. Brumlik, Srilakshmi Pandeswara, Sara M. Ludwig, Kruthi Murthy, Tyler J. Curiel

**Affiliations:** Department of Medicine, School of Medicine, and Program in Immunology and Microbiology, Graduate School of Biomedical Sciences, University of Texas Health Science Center, San Antonio, TX 78229, USA

## Abstract

Protozoan pathogens are a highly diverse group of unicellular organisms, several of which are significant human pathogens. One group of protozoan pathogens includes obligate intracellular parasites such as agents of malaria, leishmaniasis, babesiosis, and toxoplasmosis. The other group includes extracellular pathogens such as agents of giardiasis and amebiasis. An unfortunate unifying theme for most human protozoan pathogens is that highly effective treatments for them are generally lacking. We will review targeting protozoan mitogen-activated protein kinases (MAPKs) as a novel drug discovery approach towards developing better therapies, focusing on *Plasmodia*, *Leishmania*, and *Toxoplasma*, about which the most is known.

## 1. General Properties of MAPKs

Virtually all eukaryotic organisms possess MAPKs, signal transduction molecules that regulate cell functions such as tissue morphogenesis, cytoskeletal rearrangements, proliferation, differentiation, survival, immune responses, and adaptation/stress-response [1–3]. *Encephalitozoon cuniculi* is the only example to date of a eukaryote apparently lacking any MAPKs [4]. The MAPK superfamily, which evolved 1.0 to 1.5 billion years ago [5], comprises proline-directed serine/threonine kinases that are classified based on the primary amino acid sequence within the catalytic domains and must possess a [TS]XX[LIVM]XT[RK] [WY]YRXPEX[LIVM] signature sequence at its core [6–8]. The phosphorylation lip (solid underline beneath the sequence above) is required for MAPK activation by upstream regulators and is contiguous with the proline-directed (P+1) peptide binding pocket (double underline beneath the sequence above), conferring substrate specificity, and is capable of being singly [(pT)XX)] or dually [(pT)X(pY)] phosphorylated in response to particular extracellular stimuli [9]. In addition, MAPKs possess 11 subdomains [5, 10] with numerous highly conserved residues required for ATP binding, phosphotransferase activity, and substrate specificity [7].

MAPKs are often controlled by highly evolutionarily conserved regulatory cascades involving sequential phosphorylation by three component modules consisting of MAPK kinase kinases (MKKKs, Ste11-like kinases) and MAPK kinases (MKKs, Ste7-like kinases), terminating in the phosphorylation of specific MAPKs [11]. Many MAPK cascades have recently been expanded to include a fourth tier involving proteins aptly termed MKKKKs (Ste20-like kinases) [12] that can either serve in a noncatalytic capacity as a scaffold to promote pathway assembly (and MKKK auto-activation) or can phosphorylate specific MKKKs [13]. Once activated, MAPKs phosphorylate a wide variety of proteins including MAPK-activated protein kinases and transcription factors, ultimately resulting in changes in gene expression [14, 15]. MAPK signaling can also have additional epigenetic effects by affecting histone modification [16].

MAPKs are grouped into subfamilies on the basis of amino acid sequence similarity, mechanism of activation, and the type of MAPK cascade to which they belong. Cyclin-dependent kinases share very high amino acid sequence identity with MAPKs [17] but generally lack a phosphorylation lip. Differences in the precise amino acid composition of the phosphorylation lip have historically been used to classify MAPKs, as outlined below. Our phylogenetic studies [18] have established, however, that homology between many short strings of amino acids found in MAPKs is of equal or greater importance when classifying MAPKs within different subfamilies.

Four conventional MAPK subfamilies exist, which are also described as “typical”, that is, capable of dual phosphorylation [8]. These conventional MAPK groups include the extracellular signal-regulated kinases (e.g., mammalian ERK1 and ERK2, possessing a TEY motif at the phosphorylation lip, [19]), c-Jun-activated kinases (e.g., mammalian JNK1, JNK2, and JNK3 (TPY motif) [20]), p38 stress-response MAPKs (e.g., mammalian p38*α*, p38*β*, p38*γ*, and p38*δ* (TGY motif), [21]), and mammalian ERK5 (big MAPK-1, BMK-1 (TEY motif) [22, 23]). ERK5 is unusual because it possesses a long carboxy-terminal extension consisting of a transactivation domain and a nuclear localization signal facilitating translocation into the nucleus upon MAPK activation [8]. Multiple isoforms of MAPKs often exist within individual cells, which can either be activated by different MKKs or can themselves phosphorylate alternate downstream substrates [24]. Additional phylogenetically distinct MAPK subfamilies are defined by categorizing distantly related MAPKs including those from plants (TEY motif), yeasts (T[EN]Y motif), and protozoans (TXY motif, where X is often D or E, but many exceptions exist) [5, 18].

Several atypical MAPK subfamilies also exist, largely representing MAPKs that can only be monophosphorylated within their activation loops. Mammalian ERK3 [25] and ERK4 [8], possessing an SEG motif in the phosphorylation lip and an RXPR motif in the substrate binding pocket, are representative members of one major subfamily of atypical MAPKs, while Nemo-like kinases (NLKs, with a T[HQ]E motif) comprise a second major atypical MAPK subfamily [26]. Greater sequence diversity exists in the phosphorylation lip of atypical protozoan MAPKs (most commonly TGH or TSH motifs) compared to metazoan MAPKs, but members of this subfamily otherwise closely resemble typical MAPKs.

Monophosphorylated human ERK2 has 10- to 100-fold less kinase activity than dually phosphorylated ERK2 [27], illustrating that dual phosphorylation (as is the case for typical MAPKs) achieves greater signal amplification and range of responses than can be achieved by monophosphorylation (as is the case for atypical MAPKs). In addition, different upstream activators can preferentially phosphorylate the threonine or tyrosine within the activation loop of typical MAPKs, allowing signals from two different origins to elicit a response [28]. Typical MAPKs are also subject to a tertiary level of control through the expression of phosphatases specific for either phosphothreonine or phosphotyrosine in the activation loop [29].

Human ERK8 (homologous to rat ERK7) represents a prototypical member of a large atypical MAPK subfamily [30]. Although these large atypical MAPKs contain a TEY motif capable of dual phosphorylation, activation of mammalian ERK8 (or ERK7) is not under the control of any known MKK family member. Instead, they are activated by autophosphorylation of their activation loops in response to conformational changes in their carboxy-terminal extensions [31]—a highly unusual feature for mammalian MAPKs. Their carboxy-terminal extensions possess a nuclear localization signal that is only exposed in the activated state, thereby facilitating MAPK translocation to the nucleus, which in turn regulates cell proliferation [32].

**Figure 1:**
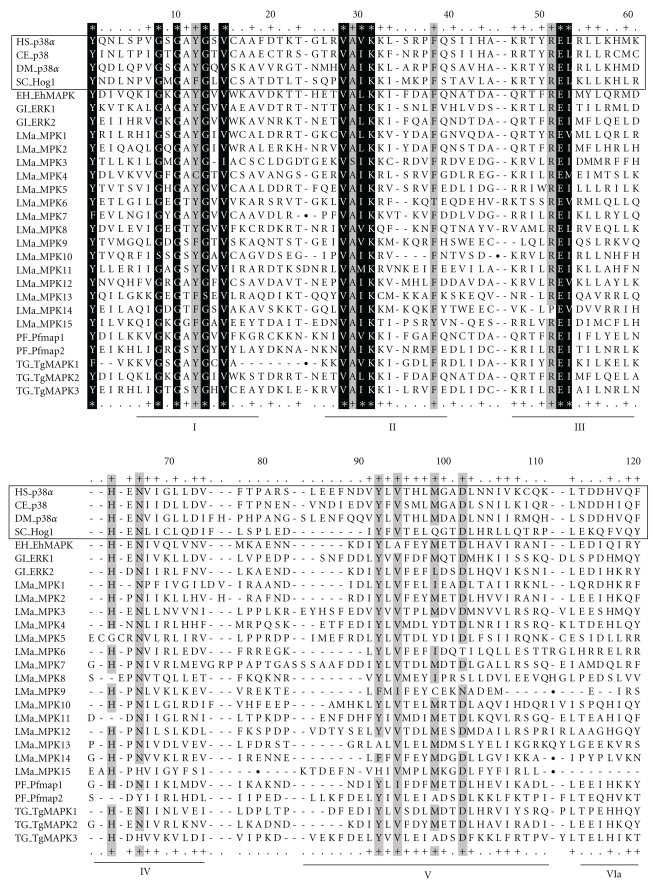

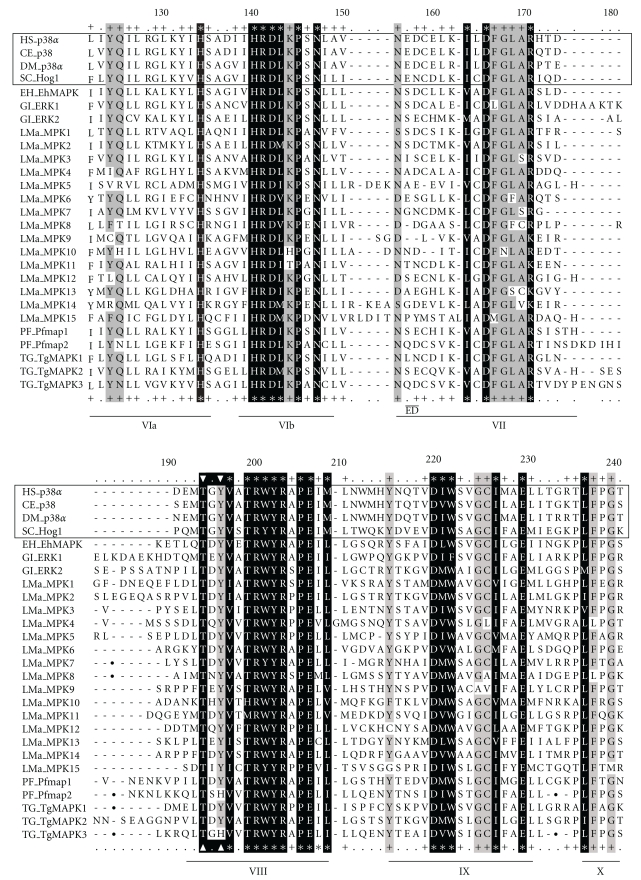

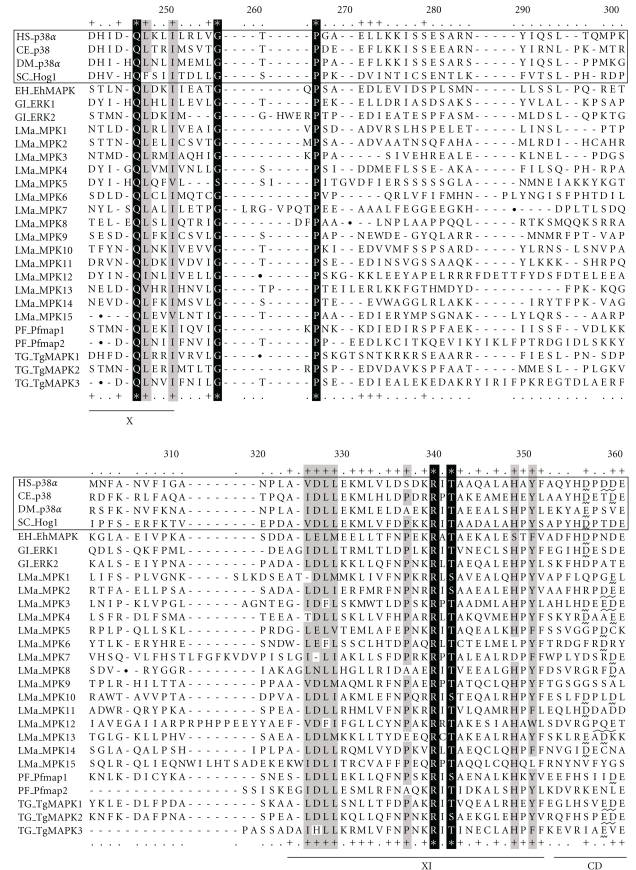

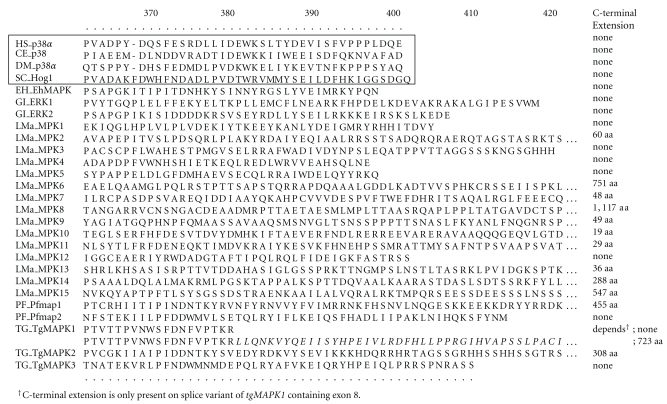
ClustalW alignment of representative MAPKs of diverse evolutionary origin, with each of the 11 subdomains indicated (Roman numerals). Conserved acidic residues within the ED site (subdomain VII) and common docking (CD) domain, which immediately follows subdomain XI, have been underlined. The first four sequences represent p38 MAPKs of metazoan and yeast origin and are boxed as reference sequences to which other protozoan MAPKs can be compared. Invariant MAPK residues (within allowed substitution groups) are highlighted in black and denoted by an asterisk. Highly conserved residues (>80% conservation) are highlighted in grey and denoted by a plus sign. In the absence of grey shading, plus signs indicate residues conserved in the majority of aligned sequences. Allowed substitution groups include acidic/amide (DE, DN, EQ), aliphatic (LIVM), aromatic (FYW), basic (KR), and hydroxyl/polar residues (STG). The positions of insertion sequences removed prior to ClustalW alignment are indicated by filled circles. White triangles denote the position of the TX[XY] phosphorylation lip. Two letter abbreviations precede the name of each MAPK sequence, indicating the genus and species of origin for each MAPK. CE: *Caenorhabditis elegans*; DM: *Drosophila melanogaster*; EH: *Entamoeba histolytica*; GI: *Giardia intestinalis*; HS: *Homo sapiens*; LMa: *Leishmania major*; PF: *Plasmodium falciparum*; SC: *Saccharomyces cerevisiae*; TG: *Toxoplasma gondii*. Accession numbers of all aligned sequences are listed in Tables 1 and 2.

We performed ClustalW alignment [33] comparing the amino acid sequences of representative metazoan (*Homo sapiens* [21, 34], *Drosophila melanogaster* [35], *Caenorhabditis elegans* [36]) and yeast (*Saccharomyces cerevisiae* [6]) p38 MAPKs to unique protozoan MAPKs described in this review (Figure 1). Human p38*α* was selected as a prototypical MAPK for comparison for three principal reasons. First, a plethora of p38 MAPK inhibitor drugs currently exists [37, 38]. Second, the binding specificity of the pyridinylimidazole p38 MAPK inhibitor SB203580 to the ATP binding pocket of human p38*α* is well understood [39, 40]. Third, we have shown that p38 MAPK inhibitors effectively inhibit the *in vitro* replication of protozoan parasites such as *Plasmodium falciparum* (Brumlik et al., submitted), *L. donovani* (Brumlik et al., unpublished observations), and *T. gondii* [41]. We have further demonstrated that the pyridinylimidazole p38 MAPK inhibitor RWJ67657 protects mice from lethal challenge with *T. gondii *[42]. Figure 1 demonstrates that while the overall structure of MAPKs is highly conserved even between distantly related eukaryotes, unique features exist that could lead to the design of MAPK inhibitors specific for protozoan parasites.

## 2. Phylum Apicomplexa

Apicomplexa is a large, diverse phylum comprising over 5000 species, of which seven are known human pathogens (in the genera *Babesia, Cryptosporidium, Cyclospora, Isospora, Plasmodium, Sarcocystis,* and *Toxoplasma*). There are no reports of functional studies of MAPKs from *Babesia, Cryptosporidium, Cyclospora, Isospora,* or *Sarcocystis* to our knowledge. This section will thus focus on *Plasmodium* and *Toxoplasma*.

### 2.1. Genus Plasmodium

The genus *Plasmodium *contains four significant human pathogens, all agents of malaria: *P. falciparum, P. vivax, P. ovale,* and *P. malariae. P. falciparum*, which causes the most severe form of malaria, possesses only two MAPKs. Its Pfmap-1 represents a typical MAPK that is predominantly expressed in gametocytes [43] while Pfmap-2 represents an atypical MAPK (Table 1, Figure 1), which instead possesses a TSH phosphorylation lip [44]. PfPK7, which bears extremely limited homology to mammalian MKK3 and MKK6 that activate host p38 MAPK, does not appear to be a true MKK homologue. Furthermore, PfPK7 is unable to phosphorylate either recombinant Pfmap-1 or Pfmap-2 *in vitro* [45], suggesting that it does not represent a long-sought-after member of an MAPK cascade in *Plasmodium*. Moreover, no *P. falciparum* MKK genes have been identified, suggesting that *P. falciparum* MAPK signaling does not utilize typical MAPK cascades [46]. *P. falciparum* Pfmap-2 is instead activated by Pfnek-1, a never-in-mitosis/*Aspergillus-* (NIMA-) related kinase [47]. Since homology amongst MKKs and MKKKs is much lower than that for members of the MAPK superfamily, it is conceivable that genes encoding these proteins exist but have simply not been annotated as such in the *P. falciparum* genome.

Pfmap-1 is neither required for schizogony nor gametocytogenesis in human erythrocytes cultured *in vitro*, nor for gametogenesis and/or sporogony in the mosquito vector [48]. However, Pfmap-2 protein levels are elevated in *pfmap-1* knockout parasites, suggesting that Pfmap-1 fulfills an important function necessitating compensatory adaptation in parasites lacking this enzyme. Pfmap-2 is essential for the completion of the *P. falciparum* asexual cycle [48]. Functional characterizations of MAPKs from *P. vivax, P. ovale,* and *P. malaria,* the other *Plasmodium* species causing malaria, have yet to be reported to our knowledge.

### 2.2. Genus Toxoplasma

*T. gondii*, the sole member of the genus *Toxoplasma*, can cause significant morbidity or mortality in hosts with compromised cellular immunity. Like *P. falciparum*, *T. gondii* appears to be another protozoan parasite that lacks typical MAPK activation cascades. Preliminary examination of the *T. gondii* genome suggests that it encodes four MAPKs. However, the TGME49_021550 locus (situated on chromosome II) lacks coding sequences corresponding to several essential MAPK motifs (including an incomplete MAPK signature sequence), thereby disqualifying it as a functional MAPK gene. Of the remaining three MAPK genes (Table 1, Figure 1), we have cloned and sequenced both the genes encoding *tgMAPK1*, situated on chromosome XI [50], and *tgMAPK2* (chromosome VIII; [18]). We have also sequenced the third MAPK gene, *tgMAPK3* (chromosome Ib).

TgMAPK1 is a critical virulence determinant during acute *T. gondii* infection (Brumlik et al., submitted). By expressing it in Hog1-deficient yeast lacking its own stress-response MAPK, we restored yeast ability to grow under osmotic stress [50], providing evidence for this MAPK's role as a stress-response MAPK. Since TgMAPK1 expression affects tachyzoite/bradyzoite stage differentiation (manuscript in preparation), we renamed it “BARKY” (bradyzoite antigen regulator, kinase Y).

BARKY is a typical MAPK based on conventional criteria [50] although it possesses three insertion sequences. Using mass spectroscopy, we confirmed the presence of a 34 amino acid insert situated between the GXGXXGXV motif (subdomain I) and the invariant lysine residue within the VAXK motif of subdomain II, a region responsible for anchoring the nontransferable *α*- and *β*-phosphates of ATP during catalysis. *BARKY* is also predicted to encode a 93 amino acid insert situated between the DFGLAR motif that interacts with the Mg^++^ bound to ATP and the phosphorylation lip, which links the proline-directed peptide binding pocket in an extended conformation following phosphorylation of its activation loop (subdomains VII and VIII, resp.). Finally, using mass spectroscopy, we identified a 20 amino acid insert between subdomains IX and X.

Phylogenetic analysis demonstrates that *T. gondii* BARKY most closely resembles *Cryptosporidium hominis* MAPK (with 52% amino acid sequence identity across all 11 of the MAPK subdomains), with a corresponding homologue in *C. parvum*. No other closely related MAPK homologues were identified either within or outside the phylum Apicomplexa at the time of publication [18].

Alternative splicing within exons 3-4 and exons 7-8 of the *BARKY* gene results in multiple BARKY isoforms, producing protein variants that could differentially respond to upstream signals or have altered substrate specificity. In support, we have detected 50, 58, and ~130 kDa proteins in *T. gondii* tachyzoite cell-free extracts by Western blotting. We have also employed mass spectroscopy to detect peptide fragments that confirm the existence of the full length (130 kDa) BARKY protein in tachyzoites grown *in vitro*. We cannot exclude the possibility that the smaller forms of the protein result from proteolytic degradation, but reverse transcriptase-polymerase chain reaction has demonstrated the presence of *BARKY* transcripts with a stop codon-situated 84 nucleotides into exon 7, as well as an alternative *BARKY* splice variant encoding exon 8 that adds a 766 amino acid extension to the carboxy-terminus (Brumlik et al., unpublished observations). These features are reminiscent of extensions identified in many *Leishmania* MAPKs [51].

There are no reported functional data for *T. gondii* TgMAPK2 but it is expressed in *T. gondii* tachyzoites at the expected molecular weight of 73 kDa (Brumlik et al., unpublished observations). Phylogenetic analysis places this MAPK in a group of closely related Apicomplexan MAPKs which includes *Cryptosporidium hominis* MAPK1, *P. falciparum* Pfmap-1, and *Theileria annulata* MAPK (all sharing roughly 70% amino acid sequence identity across all 11 of the MAPK subdomains). TgMAPK2 shares significant amino acid sequence identity with MAPKs from non-Apicomplexan protozoans including *L. mexicana* LmxMPK2 (62%) and *Trypanosoma brucei* TbMAPK2 (62%), each possessing a typical TDY phosphorylation lip. The deduced amino acid sequence of TgMAPK2 shares 55% identity with human ERK8 across all 11 MAPK subdomains, demonstrating the remarkable evolutionary conservation of this MAPK subfamily member. In addition, *T. gondii* TgMAPK2 possesses multiple copies of a VSSSHHG repeat in its carboxy-terminal extension, the exact number of repeats being strain-dependent [18]. While the role of this repeat remains unknown, it is striking that *P. falciparum* Pfmap-1 possesses an analogous series of imperfect KKYVD[GSE][GSL]N repeats in its carboxy-terminal extension [43]. Short amino acid repeats often facilitate oligomerization or serve as contact points for protein-protein interactions. Interestingly, TgMAPK2 is also predicted to possess a nuclear localization signal within its carboxy-terminal extension.

*T. gondii* TgMAPK3 is predicted to be an atypical 63 kDa MAPK with a TGH phosphorylation lip. It shares significant amino acid sequence identity with several Apicomplexan MAPKs such as *Cryptosporidium hominis* MAPK2 (67%), *P. falciparum* Pfmap-2 (58%), and *Theileria annulata* MAPK2 (50%), with low amino acid sequence identity to non-Apicomplexan MAPKs [18].

## 3. Phylum Sarcomastigophora

### 3.1. Trypanosomatid MAPKs

Trypanosomatids (members of the family Trypanosomatidae) are a diverse group of protozoan parasites of which two genera are human pathogens: *Trypanosoma* and *Leishmania*.

#### 3.1.1. Genus Leishmania

Several different *Leishmania* species cause human disease of varying clinical presentation and severity, of which *L. major* generally causes the most serious illnesses. Genome sequencing has identified 15 putative complete MAPK genes in *L. major* (Table 2), the alignments of which are shown in Figure 1. Two partial *L. major* MAPK genes have also been identified (LmjF03.0210 and LmjF13.07800) [52] but have been excluded from further consideration because they lack the coding region for the complete MAPK signature sequence. All 15 *L. major* MAPK homologues have also been identified in *L. mexicana* (Table 2), *L. infantum*, and *L. brasiliensis* [52].

Each of the 15 unique *Leishmania* MAPKs (Figure 1) is a typical MAPK by the classical definition (*i.e.*, the activation loop is comprised of a TXY motif). The majority of these MAPKs possess carboxy-terminal extensions (Figure 1), some of them over 1000 amino acids long (as for LmaMPK8). This region may be analogous to the corresponding region of human ERK5 or ERK8, each of which possesses a C-terminal transactivation domain and nuclear localization signal [22, 23]. LmaMPK6, 7, and 8 are predicted to contain nuclear localization signals within their carboxy-terminal extensions, making them even more closely resemble human ERK5 and ERK8, as well as *T. gondii* TgMAPK2.

Deletion analysis of the genes encoding *L. mexicana* LmxMPK1 and LmxMPK2 demonstrates that both are essential for amastigote (bloodstream stage) survival [52, 53]. *L. mexicana* LmxMPK4 is essential to both promastigote (sandfly stage) and amastigote forms [58] and is phosphorylated on T^190^ and Y^192^ of its phosphorylation lip by the MKK LmxMKK5 [64]. Overexpression of *L. major* LmaMPK4, 7, or 10 (homologues of LmxMPK4, 7, and 10, resp.) causes stage-specific induction of phosphotransferase activity. Moreover, LmaMPK7 activation specifically regulates parasite growth [62]. In each case, kinase activity was low or absent in cell-free extracts from promastigotes but significantly increased after exposure to pH 5.5 and 34°C., which simulates the stress encountered by the parasite in the acidified phagolysosome upon invasion of macrophages [59]. *L. mexicana* LmxPK4 is an MKK that controls parasite differentiation [65] and thus represents a potential upstream activator of at least one of the MAPKs affecting stage differentiation.

Several *L. mexicana* MAPKs regulate flagellar length, many of which possess carboxy-terminal extensions [66]. Deletion mutants for LmxMPK3 had shortened flagella and overexpression of LmxMPK3 in the deletion background complemented this defect [56, 57]. Deletion mutants for LmxMPK9, LmxMPK13, or LmxMPK14 generated promastigotes with elongated flagella, an effect that could be reversed by overexpressing these MAPKs in null mutants [57, 63]. LmxMPK13 is the homologue of LF4 from the protozoan microalga *Chlamydomonas reinhardtii*, which also regulates flagellar length [67]. *L. mexicana* LmxMKK is the MAPKK responsible for regulating flagellar length [68] and activates LmxMPK3 [56] and perhaps affects other MAPKs regulating flagellar length.

Analysis of the *L. mexicana* genome has identified two additional putative MKK genes in addition to *L. mexicana lmxPK4*, *lmxMKK,* and *lmxMKK5* for which functions have yet to be determined. *L. mexicana* also putatively encodes 23 MKKKs and a single MKKKK [51], the functions of which remain unknown.

#### 3.1.2. Genus Trypanosoma

Subspecies of *T. brucei* cause African sleeping sickness, whereas *T. cruzi* causes New World trypanosomiasis (Chagas disease). Genomic sequencing has identified 13 MAPK genes in *T. brucei*, each of which has at least one, but often two virtually identical copies of MAPK homologues in *T. cruzi* (with each copy having greater than 99% amino acid sequence identity to the other (Table 2)) [51, 52]. Homologous *T. brucei* or *T. cruzi* MAPK domains are ~90% identical to each other and each has a single corresponding homologue in *Leishmania* spp. (sharing over 80% amino acid sequence identity across the 11 MAPK subdomains). LmxMPK7 and LmxMPK8 are the only two *Leishmania* MAPKs that lack homologues in either *T. brucei* or *T. cruzi*. Thus we exclusively used the *L. major* MAPK sequences (LmaMPK1-15) for ClustalW alignment, reducing redundant examples of highly homologous MAPKs in the analysis.

Although all *Trypanosoma* MAPKs possess a classical TXY motif, a feature also conserved in all *Leishmania* MAPK homologues (Table 2), the central amino acid in the TXY motif varies between MAPK homologues from different Trypanosomatid species (Table 2) and thus is not as evolutionarily constrained as in mammalian MAPKs.

*T. brucei/cruzi* MPK10 (accession nos. Q580Z7/Q4D4Q4) and MPK11 (accession nos. Q389D8/Q4CZQ7) have not yet been officially named (see Table 2). Regardless, these MAPKs (and their *Leishmania* MAPK homologues) are exceptional in possessing a MAPK signature sequence that deviates with respect to the precise position of the threonine in the proline-directed (P+1) peptide binding pocket (see Figure 1, center of subdomain VIII, residues 197-207). This likely alters the precise spatial orientation of the proline-directed peptide binding pocket relative to the phosphorylation lip, perhaps placing these MAPKs in a separate subfamily.

KFR1 (KSS1- and FUS3-related kinase 1), the *T. brucei* homologue of *L. mexicana* LmxMPK1, mediates interferon-*γ*-induced amastigote proliferation and phosphorylates serine residues on host histone H1, myelin basic protein, and *β*-casein [54, 55]. *T. brucei* TbECK1, which is the trypanosome homologue of *L. mexicana* LmxMPK6, possesses a carboxy-terminal extension that regulates kinase activity in all life cycle stages. Expression of a truncated TbECK1 protein lacking large parts of this extension caused *T. brucei* to grow slowly with abnormal morphology [61]. *T. brucei* procyclic forms lacking TbMAPK5, the homologue of *L. mexicana* LmxMPK5, likewise showed impaired differentiation into the bloodstream form [60]. TbMAPK2, the *T. brucei* homologue of LmxMPK4, regulates cell cycle progression from the procyclic (tsetse fly midgut) form to the bloodstream form [69]. TbMAPK5 controls *T. brucei* differentiation [60]. No functional studies of *T. cruzi* MAPKs have been published to date to our knowledge.

Phylogenetic analysis of *T. brucei* and *T. cruzi* suggests a single gene orthologous to the five putative MKK genes in *L. major* [51]. Only about one-third of the putative *L. major* MKKK genes have phylogenetic branching patterns consistent with the existence of orthologous genes in *T. brucei* and *T. cruzi* [51]. In most cases, *L. major* and *T. brucei* MKKK genes appear to be paralogues, having arisen from gene duplication events [51], suggesting significant evolutionary divergence in the circuitry of signaling cascades in Trypanosomatids. Three unique MKKKK genes have been identified in *T. cruzi* and two in *T. brucei* [51]. Functions have yet to be ascribed to any of these putative upstream MAPK activators.

### 3.2. Other Sarcomastigophora

Two MAPKs have been identified and characterized in the protozoan intestinal parasite *Giardia lamblia*, ERK1 and ERK2 (Table 1, Figure 1), each of which plays distinct roles in encystation [49]. In addition, one MAPK gene has been identified in the *Trichomonas vaginalis* genome [70]. However, functional studies have yet to be performed on MAPKs from either parasite.

### 3.3. Subphylum Sarcodina (the Amoebae)

This subphylum of amoebas contains three human pathogenic genera: *Entamoeba*, *Naegleria, and Acanthamoeba*. The *E. histolytica EhMAPK* gene encodes a putative MAPK with significant homology to human ERK8 [71]. We are not aware of any further MAPK analyses in this genus or of any reports of MAPK genes or function in *Naegleria* or *Acanthamoeba*.

## 4. Protozoan MAPKs as Therapeutic Targets

MAPKs direct many functions critical to pathogen homeostasis and survival, including proliferation [62], differentiation [52, 53], regulation of cytoskeletal features such as the biosynthesis of flagella [56, 57, 63], and stress-responses [50]. Because protozoan MAPKs share many common structural features and are vastly more closely related to each other than to human MAPKs [18, 72], it should be possible to design drugs specifically or preferentially targeting protozoan MAPKs. For example, *Leishmania mexicana* LmxMPK1 and LmxMPK2 are essential MAPKs required for differentiation [52, 53], with corresponding homologues in other *Leishmania* species and in *T. brucei* and *T. cruzi* [51], but bearing scant resemblance to human MAPKs, making them excellent candidates for drug development [72]. Specifically targeting these MAPKs could have far reaching therapeutic potential since one drug could be used to treat a broad range of Trypanosomatid infections based on the high degree of homology between Trypanosomatid MAPKs [51].

*P. falciparum* Pfmap-2 is likewise an excellent druggable target as this MAPK is essential for the parasite to complete asexual replication in infected human erythrocytes [48] and it is highly dissimilar to human MAPKs. Although we have yet to determine which of the *T. gondii* MAPKs are essential to parasite survival, reducing BARKY expression dramatically impairs parasite virulence (Brumlik et al., submitted), making BARKY a useful target for MAPK inhibitor drugs.

Agents interfering with the function of MAPKs that affect stage differentiation, such as *T. gondii* BARKY, or affect parasite growth, such as *L. major* LmaMPK7 or *T. brucei* TbECK1, likely would be useful antiparasitic agents. *T. brucei* KFR1 is an interesting MAPK target, as it regulates effects of the host immune response (interferon-*γ*-induced amastigote proliferation) and could be considered in combination with an immune strategy. *L. mexicana* LmxMPK1 is homologous to KFR1 and could mediate similar effects, being a useful drug discovery target in this respect. Agents impairing the function of MAPKs controlling flagellar development or function, such as LmxMPK3, LmxMPK9, LmxMPK13, or LmxMPK14, could inhibit parasite dissemination and might be useful alone, or in combination with parasiticidal agents.

Our work with *T. gondii* BARKY demonstrates multiple MAPK splice variants that can occur naturally in parasites. A better understanding of the function of these splice variants could help develop agents specifically targeting variants relevant to disease pathogenesis. Likewise, our genomic analyses, and those of others, have demonstrated unusual repeat motifs in several protozoan parasite MAPKs (including in *T. gondii* and *Plasmodium* species) encoding large numbers of potential phosphorylation sites. An understanding of the functional significance of these motifs could help develop useful antiparasitic agents. Given the relatively unique nature of the phosphorylation site repeat motifs, these sites possibly could lead to highly parasite-specific drugs.

Protozoan MAPKs need not subserve critical functions to be useful drug discovery targets. For example, *L. mexicana* LmxMPK6 affects parasite morphology (which has indirect consequences on its growth rate following infection) and has homologues in related disease-causing Trypanosomatids. Drugs impairing LmxMPK6 function could be used in conjugation with existing anti-*Leishmania* therapies to boost their efficacy and could have broad-spectrum effects.

Upstream components of the MAPK cascades such as the MKKs or MKKKs in pathogenic protozoan parasites are also potentially useful drug discovery targets. For example, the *L. mexicana* MKK, LmxPK4, controls parasite differentiation and thus is an excellent candidate. Because protozoan MKKs and MKKKs are even more distantly related to mammalian counterparts than MAPKs, a further potential advantage to this approach is that drugs inhibiting parasite MKK function could be less likely to have undesirable side-effects compared to drugs targeting specific MAPKs.

A potential disadvantage to targeting upstream MAPK regulators relates to our incomplete understanding of how they function. For example, MAPKs such as human p38*α* are capable of MKK-independent activation and can undergo autophosphorylation in the presence of transforming growth factor-*β*-activated protein kinase 1 [73]. In this case, it would not be possible to block p38*α* activation by targeting the conventional upstream MKKK and MKK components of the p38 MAPK cascade.

Many protozoan MAPKs possess vestiges of the common docking (CD) domain and ED site (Figure 1)—surface-exposed acidic residues in human p38*α* MAPK that facilitate binding to upstream and downstream MAPK partners [74, 75]. D^313^, D^315^, and D^316^ comprise the CD domain in human p38*α* MAPK. This region acts in concert with the ED^161^ site to bind to short strings of 2–5 basic amino acids situated on proteins with which p38*α* interacts [74]. Protozoan MAPKs lacking a conserved CD domain (e.g., *Leishmania major* LMaMPK9 and 15) and/or ED site (e.g., *Leishmania major* LMaMPK3, 7, 9, 11, 15, and *T. gondii* TgMAPK1) are prime candidates for drug development since these domains have diverged considerably from their corresponding mammalian counterparts.

In addition, the highly variable carboxy-terminal extensions, which are present in over half the protozoan MAPKs shown in Figure 1, are excellent targets for drug development owing to their unique structures. Drugs targeted to these extensions would have a low probability of affecting mammalian MAPKs.

SB203580 is a pyridinylimidazole competitive ATP inhibitor affecting human p38 MAPK phosphotransferase activity through hydrogen bonding between its pyridine ring nitrogen and the MAPK backbone amide of M ^109^ in the THLM^109^ motif (subdomain V; Figure 1) [39]. A second critical hydrogen bond occurs between a nitrogen atom on the imidazole ring and the invariant lysine in the VAXK ^53^ motif (subdomain II). Finally, the fluorophenyl ring of SB203580 interacts with the hydrophobic environment created by T^106^ and H^107^[39].

Because SB203580 is much smaller than ATP (as are all pyridinylimidazole p38 MAPK inhibitors), it does not fully occupy this region, leaving two large hydrophobic pockets on either side of the pyridine ring [39]. By designing novel pyridinylimidazoles or structurally related pharmacophores that properly fill the ATP binding pocket of pertinent protozoan MAPKs, it could be possible to develop novel antiparasitic agents that are more potent and specific than existing drugs. Such drugs will be less likely to have unintended consequences on host p38 MAPK, which is a potential drawback of several existing p38 MAPK inhibitors.

Recent molecular modeling studies using competitive ATP inhibitors against LCRK3 in *L. donovani*, a cyclin-dependent kinase that is a distant relative of the MAPK superfamily, indicate that such compounds could have significant inhibitory activity against *L. donovani* LCRK3 [76]. Our work has shown that the human p38 MAPK inhibitors RWJ67657, RWJ68198, and SB203580 reduced the replication of *L. donovani* promastigotes in axenic culture. Moreover, SB203580 effectively inhibited the replication of the bloodstream stage cultured *ex vivo* (Brumlik et al., unpublished observations).

X-ray crystallographic studies of human p38*α* MAPK complexed with ATP have demonstrated that the THLM^109^ motif in the center of subdomain V (Figure 1) forms two critical hydrogen bonds with the adenosine moiety [77]. Based on our ClustalW alignment, many other amino acids can evidently serve this same purpose in other MAPKs (Figure 1; subdomain V), although the binding affinity of ATP (and competitive ATP inhibitor drugs) could be affected by such differences. Structural studies have further shown that the invariant GXGXXGXV^38^ motif in subdomain I coordinates the nontransferable *α*- and *β*-phosphates of ATP, while catalytic transfer of the *γ*-phosphate is mediated by hydrogen bonding between an essential lysine in the VAXK ^53^ motif (subdomain II), the RE^68^ motif in subdomain III, and the underlined residues in the HRD ^168^XK ^170^PXN ^173^ motif (subdomain VIb) [78]. Thus, to design novel competitive ATP inhibitors against protozoan MAPKs, one must not only account for the invariant residues comprising the ATP binding site in all MAPKs but also pay particular attention to the permissible structural changes in subdomain V of protozoan MAPKs that specifically affect the binding of competitive ATP inhibitors.

We have shown that SB203580 [50] and another pyridinylimidazole human p38 MAPK inhibitor, RWJ67657, significantly inhibit BARKY autophosphorylation (Brumlik et al., unpublished observations). These agents reduced *T. gondii* proliferation *in vitro* [41] and treated otherwise fatal *T. gondii* infection in mice [42]. We further assessed the efficacy of two human p38 MAPK inhibitors to treat parasitic infections and showed that RWJ67657 and the pyrrolobenzimidazole RWJ68198 effectively blocked the replication of *P. falciparum* cultured in human erythrocytes *ex vivo*. Drug treatment resulted in trophozoites that were markedly diminished in size (Brumlik et al., submitted).

We demonstrated that RWJ67657 protected mice from otherwise fatal infection with the protozoan *Encephalitozoon cuniculi* [42] although it encodes no known MAPKs. Inhibition of host p38 MAPK could improve the host immune response to *E. cuniculi*, as has been demonstrated for *T. gondii* [79], or RWJ67657 could have therapeutic off-target effects in either the host or parasite. Better understandings of the mechanism of action in this case will further help drug development.

A large number of p38 MAPK inhibitors have recently progressed into phase I and II clinical trials, thus providing basic inhibitor pharmacophores that can be modified to target critical protozoan MAPKs specifically while at the same time having less host toxicity (a problem with many agents in the pyridinylimidazole class).

## 5. Conclusions

MAPKs play essential roles in virtually all eukaryotes. Thus, inhibiting protozoan MAPK functions represents a scientifically sound approach to developing novel classes of antiprotozoan agents. As protozoan MAPKs are only distantly related to mammalian MAPKs and have distinct active sites, it is reasonable to expect that selective agents can be developed to target pathogen proteins with minimal collateral effects on human counterparts.

Although only a very modest body of work on the structure and function of protozoan MAPKs currently exists, the available evidence already suggests the general utility of inhibiting protozoan parasite MAPK function as a treatment strategy. Several specific MAPK candidates have also already emerged from such work. As interest in MAPKs increases, the rate of important discoveries and their preclinical and clinical translation will also increase.

Specific roles for MAPKs cannot be predicted based solely on sequence similarity to protein homologues. For example, *P. falciparum* MAPK Pfmap-2 is essential for the completion of asexual replication in human erythrocytes [48] and yet its closest homologue in *P. berghei*, Pbmap-2 (with 93% amino acid sequence identity within its catalytic domains to Pfmap-2 [80]), is dispensable for both asexual replication and gametocyte formation in the mouse erythrocyte. Pbmap-2 instead plays a critical role in exflagellation in the mosquito midgut [81]. Thus, while the structure of the MAPKs themselves remains highly evolutionarily constrained even among closely related *Plasmodium* species, the circuitry of the various signal transduction pathways themselves has undergone significant divergent evolution. Therefore it is critical to establish specific roles of particular MAPKs prior to drug development.

Once the function of a MAPK from a pathogenic protozoan parasite has been established, one can exploit the phylogenetic differences between MAPKs of protozoan and metazoan origin to design specific MAPK inhibitors. Refining the structure of human MAPK inhibitor pharmacophores already in existence should speed development of new MAPK-inhibiting antiprotozoan drugs. We also expect to see additional new classes of drugs developed, which will be aided by additional structure/function studies. Targeting upstream MAPK regulators is an approach that also bears investigation, but which will likely lag owing to significant current knowledge gaps in understanding these regulators.

A considerable challenge is to persevere with such research given the relatively scant resources available for such work, in spite of the fact that over one-half billion people in many of the poorest parts of the world are infected by pathogenic protozoan parasites [82].

## Figures and Tables

**Table 1:** Non-Trypanosomatid mitogen-activated protein kinases discussed in this review.

Organism	MAPK	Accession no.	Phosphorylation lip	Classification	Function	References
*Caenorhabditis elegans*	p38	AAB00664	TGY	Typical	Stress-response	[36]
*Drosophila melanogaster*	p38*α*	AF035547	TGY	Typical	Stress-response	[35]
*Entamoeba histolytica*	EhMAPK	AY460178	TDY	Typical	?	
*Giardia intestinalis*	ERK1	AY149274	TEY	Typical	Encystation	[49]
	ERK2	AY149275	TDY	Typical	Encystation	[49]
*Homo sapiens*	p38*α*	Q16539	TGY	Typical	Stress-response	[21, 34]
*Plasmodium falciparum*	Pfmap-1	Q94656	TDY	Typical	?	
	Pfmap-2	Q25917	TSH	Atypical	Essential for differentiation	[48]
*Saccharomyces cerevisiae*	Hog1	AAA34680	TGY	Typical	Stress-response	[6]
*Toxoplasma gondii*	TgMAPK1 (BARKY)	AY684849	TDY	Typical	Proliferation^†^, differentiation^†^, virulence^†^	
	TgMAPK2	DQ115400	TDY	Typical	?	
	TgMAPK3	XP_0022369585	TGH	Atypical	?	

^†^Brumlik et al., submitted.

**Table 2:** Mitogen-activated protein kinases and their corresponding homologues in Trypanosomatids.

*L. major* (LMa) MAPK	LMa ^a^accn.	*L mexicana* (LMx) MAPK	LMx ^a^accn.	*T. brucei* (TB) MAPK	TB ^a^accn.	*T. cruzi* (TC) MAPK	TC ^a^accn. #1	TC ^a^accn. #2	Phosphorylation lip LMa/LMx/TB/ TC#1/TC#2	Function	References
LmaMPK1	Q4Q0B0	LmxMPK1	Z95887	KFR1	Q26802	—	Q4CSB9	—	TDY/TDY/ TEY/TGY	Essential for intracellular parasite survival of bloodstream stage (LMx, TB), IFN-*γ*-induced proliferation of bloodstream stage (TB)	[52–55]
LmaMPK2	Q4Q204	LmxMPK2	AJ293280	—	Q38B88	—	Q4CZ09	Q4CR01	TDY/TDY/TDY/ TDY/TDY	Essential for intracellular parasite survival of bloodstream stage (LMx, TB)	[52, 53]
LmaMPK3	Q4QHG6	LmxMPK3	AJ293281	—	Q580X5	TcMPK3	Q4D0A7	Q4CKS6	TDY/TDY/TDY/ TDY/TDY	Flagellar length (LMx)	[56, 57]
LmaMPK4	Q4QD66	LmxMPK4	AJ293282	TbMAPK2	Q38B88	—	Q4D3Y2	—	TQY/TQY/ TDY/TEY	Stage-specific induction of phosphotransferase activity(LMx)	[52, 58, 59]
LmaMPK5	Q4Q701	LmxMPK5	AJ293283	TbMAPK5	Q586Y9	—	Q4DHF7	Q4DCP6	TDY/TDY/TDY/ TDY/TDY	Differentiation (TB)	[60]
LmaMPK6	Q4Q4U7	LmxMPK6	AJ293284	TbECK1	Q381A7	—	Q4DD40	—	TDY/TDY/ TEY/TDY	Proliferation; stage-specificinduction of phosphotransferase activity (TB)	[61]
LmaMPK7	Q4QFZ0	LmxMPK7	AJ293285	—	—	—	—	—	TDY/TDY	Proliferation; stage-specificinduction of phosphotransferase activity (LMa)	[59, 62]
LmaMPK8	Q4Q8L2	LmxMPK8	AJ293286	—	—	—	—	—	TNY/TNY	?	
LmaMPK9	Q4QDK3	LmxMPK9	AJ293287	—	Q387N8	—	Q4DYK0	Q4DD15	TEY/TEY/TEY/ TEY/TEY	Flagellar length (LMx)	[57, 63]
LmaMPK10	Q4QHJ8	LmxMPK10	DQ308411	—	Q580Z7	—	Q4D4Q4	Q4CU32	THY/THY/THY/ THY/THY	Stage-specific induction of phosphotransferase activity (LMa, LMx)	[59, 62]
LmaMPK11	Q4Q449	LmxMPK11	DQ026027	—	Q389D8	—	Q4CZQ7	Q4DC97	TDY/TDY/TDY/ TDY/TDY	?	
LmaMPK12	Q4Q7S2	LmxMPK12	DQ026026	TbMAPK4	Q585N3	—	Q4DHP2	—	TQY/TQY/TSY/THY	?	
LmaMPK13 (LF4)	Q4FVX2	LmxMPK13 (LF4)	DQ812905	MOK	Q38E60	—	Q4E4I5	Q4DWW0	TEY/TEY/TEY/ TEY/TEY	Flagellar length (LMx)	[57]
LmaMPK14	Q4FYW2	LmxMPK14	DQ812906	—	Q57WV2	—	Q4D0S5	Q4D7J6	TDY/TDY/TDY/ TDY/TDY	Flagellar length (LMx)	[57]
LmaMPK15	Q4Q3Y0	LmxMPK15	DQ812907	—	Q389P3	—	Q4DKI1	—	TIY/TIY/TFY/TFY	?	

^a^accn.; accession no.
